# Evaluation of the Performance of Haemoglobin Colour Scale and Comparison with HemoCue Haemoglobin Assay in Diagnosing Childhood Anaemia: A Field Validation Study

**DOI:** 10.1155/2019/3863070

**Published:** 2019-07-01

**Authors:** Maduka Donatus Ughasoro, Anazoeze Jude Madu, Iheoma Clara Kela-Eke

**Affiliations:** ^1^Department of Paediatrics, University of Nigeria, Enugu Campus, Enugu, Nigeria; ^2^Department of Haematology, University of Nigeria, Enugu Campus, Enugu, Nigeria; ^3^Department of Haematology, University of Nigeria Teaching Hospital, Ituku/Ozalla, Enugu, Nigeria

## Abstract

**Background:**

Anaemia in children has high mortality. We present the results of assessment of the accuracy of Haemoglobin Colour Scale in identifying anaemia compared with HemoCue assay.

**Methods:**

The presence of anaemia in 524 children from four communities was screened using the Haemoglobin Colour Scale (HCS) and HemoCue assay. Independent healthcare providers that estimated the haemoglobin level using Hb-301 haemoglobinometer were different from those that read the colour scale. The sensitivity, specificity, positive predictive value, and negative predictive value were estimated.

**Results:**

Of the 524 children surveyed, 44.5% (233/524), 50% (262/524), and 32.2% (168/524) were found to be anaemic using the HemoCue, HCS (*p*= 0.25), and clinical pallor (*p*=0.03) respectively. Using the HemoCue as standard, the sensitivity of the HCS and clinical pallor was 89.1% and 72.1%, respectively, and specificity 90.2% and 84.6%, respectively. 74.7 % of the colour scale result was within the 1.0g/dl of the HemoCue reading and 23 % was within 2.0g/dl.

**Conclusion:**

The HCS can improve the ability to detect anaemia especially where the use of the HemoCue is not feasible as in the resource poor countries. However, every case of anaemia requires further investigation to determine the underlying causes.

## 1. Background

A high proportion of the global population are anaemic [1] and children are the worse affected [1, 2]. Over the past decades, in addition to clinical detection of anaemia [3], several innovatory simple and reliable point-of-care diagnostic tests and methods for estimating haemoglobin have been introduced to aid detection of anaemia [2–7] in both developed and developing countries. In developing countries, with the high prevalence of anaemia, the screening for childhood anaemia is often limited to clinical inspection of different sites: conjunctiva, nail bed, palm of the hand, and buccal mucosal with varied outcome [8].

It is over a decade since WHO introduced the Haemoglobin Colour Scale (HCS) which requires only a drop of blood on a special paper compared with a chart of different shades of red colour and very suitable for resource poor countries [9, 10]. But a systematic review by Critchley and Bates revealed varied sensitivity and specificity of the HCS in detecting anaemia. Most of the studies reported good outcome and recommended the use of the HCS [9, 11], while few reported that the HCS is too inaccurate for general use, particularly places where the HemoCue haemoglobinometer is available [12]. Unfortunately, deployment of the HemoCue haemoglobinometer in sub-Saharan areas (SSA) has being enshrouded with challenges: scarcity and the high cost of HemoCue cuvettes and reliance on a power source (either battery or electricity).

It is over a decade since the HCS was introduced and it is rarely used in paediatric practice especially on children under 5 years old, in Nigeria and most SSA. Currently, there is a paucity of studies on the validity of the HCS on children population [13, 14] and our literature search revealed that none of such studies has been conducted in Nigeria.

In this study, we evaluated the diagnostic accuracy of the HCS, among children, implemented by different healthcare workers located in the urban and rural health facilities compared with the validated digital HemoCue analyzer using a standard protocol. The outcome provided evidence required for policy reform in the use of HCS on children in Nigeria.

## 2. Methods

### 2.1. Ethical Consideration

The University of Nigeria Teaching Hospital Enugu, Health Research and Ethics Committee, gave approval for the study. The parents/caregivers of the children gave written consent before their children were allowed to participate in the study. Anaemic children were given haematinic and referred to paediatric clinic for further evaluation.

### 2.2. Study Design

The study was a double blinded field validation of the HCS conducted in four communities randomly selected from Enugu and Abia states, both in southeast Nigeria.

### 2.3. Sample Size

A minimum sample size of three hundred and seventy-four children was estimated based on sensitivity and specificity of clinical pallor of 70%, respectively [15], 95% confidential interval, and anaemia prevalence of 42%.

### 2.4. Patient Recruitment

Stratified multistage cluster sampling was used to select the wards where the study took place. One local government area (LGA) was randomly selected from the list of LGAs in each of the study states. In the selected LGA, one rural ward and one urban ward were selected using the simple random sampling. A cluster comprises streets in the urban area and hermits in the rural areas. The households with children under 10 years of age were identified and enumerated. The households to participate were randomly selected from the list of the enumerated households. Consent to participate was obtained from the head of the household or their representative. A household was defined as a family unit “eating from the same pot”. Those who gave their consent to participate in the study were invited to the designated study health centre in their locality.

### 2.5. Data and Sample Collection

A standard protocol was followed for blood collection. The pulp of the finger or the heel of the foot was cleaned with alcohol solution. The skin was punctured with a lancet and allow free flow of blood, not squeezed on the cuvette of the HemoCue 301 machine for the haemoglobin estimation and one filter paper for the HCS.

Haemoglobin Colour Scale: Two staff members of the designated health centres were trained for 2 days on the use of the HCS (CoPark, Hamburg, Germany) which has six standard shades of red colour corresponding to haemoglobin levels of 4g/dl, 6g/dl, 8g/dl, 10g/dl, 12g/dl, and 14g/dl. To determine the haemoglobin level using the HCS, a single drop of blood was placed on an absorbent paper test strip, matched against the colour scale standards, and viewed through a circular window on the colour shade until a matching colour which corresponds to Hb value is determined. There is provision for intermediate odd values, e.g., 5g/dl, 7g/dl, 9g/dl, 11g/dl, and 13g/dl, if the healthcare worker judged the colour of the drop of blood on the test strip to fall between two standards. This assessment was done in well illuminated place during day light to reduce the effect of poor lightening or coloured light on the readings.

Another independent researcher, blinded to the haemoglobin level estimated using HCS, estimated the haemoglobin level using a validated HemoCue 301 machine. All the procedures of blood collection adhered to the World Health Organization (WHO) standard of universal precaution [16].

### 2.6. Data Analysis

The data was entered in SPSS version 20. The children were grouped as not anaemic based on WHO cutoff of ≥ 11g/dl, and anaemic if Hb level was less than 11g/dl [17]; in this study, sensitivity and specificity of the HCS were based on comparing the haemoglobin results with those of the HemoCue readings. The results were matched to determine the extent of closeness or discrepancies of the HCS to HemoCue reading. Sensitivity is true positive/true positive plus false negative, and specificity is true negative/true negative plus false positive. True positives were those children whose HCS reading was the same as the HemoCue reading or varied with ± 1g/dl, HemoCue Hb reading. True negatives were those whose HCS estimate differed from the HemoCue Hb reading by ≥ 2g/dl, being either higher or lower. False positives were those whose HCS estimate was 1.1g/dl but < 2g/dl more than the HemoCue Hb estimate. False negatives were those whose HCS estimate is 1g/dl but < 2g/dl less than the HemoCue Hb estimate. The significant value was p ≤0.05.

## 3. Results

Out of 573 children who were involved in the study, 524 children had complete data and were included in the analysis. Of the 524 children under 10 years included in the analysis, 307 children were from urban areas (167 from Abakpa, Enugu; 140 from ward 1 Umuahia central, Umuahia) and 216 children were from rural areas (106 from Ibagwa, Enugu; 110 from Nkwoegwu, Umuahia). The numbers of male and female children were 297 (56.7%) and 226 (43.3%), respectively. The mean age was 46.65 (± 34.2) months.

Using HemoCue, 233 (233/524, 44.5%) children were found to be anaemic compared to 262 (262/524, 50%) children found to be anaemic using HCS, and the difference was not statistically significant (*p* = 0.25). The sensitivity, specificity, positive predictive value, and negative predictive value of the HCS for detection of anaemia compared to HemoCue 301 as the standard were represented in Table 1. HCS had sensitivity of 89.1% and specificity of 90.2%. The positive predictive value (PPV) and negative predictive value (NPV) of HCS were 89.1% and 90,2% respectively.

Using HCS, 233 (233/524, 44.5%) children were found to be anaemic compared to 168 (168/524, 32.2%) children found to be anaemic based on clinical assessment, and the difference was statistically significant (*p* = 0.03), Table 1. The sensitivity, specificity, positive predictive value, and negative predictive value of clinical detection of anaemia compared to HemoCue as the standard were 72.1%, 84.6%, 72.1%, and 84.6%, respectively.

The proportion of HCS results that differed from the HemoCue assay with ± 1g/dl, 1.1 g/dl but ≤ 2g/dl, and > 2g/dl were 74.7%, 23%, and 2.3%, respectively. See Table 2.

## 4. Discussion

The prevalence of childhood anaemia was high and is similar to what has been reported [18, 19]. This has exceeded the 40% cutoff for severe category according to the WHO classification of anaemia in a population based on the prevalence estimated from blood levels of haemoglobin [2, 20]. By this current high prevalence, compared to the previous WHO estimates [19], Nigeria has not made much impact on its fight to control anaemia, at least in the region where the study was conducted. Thus, there is a need to reevaluate the existing anaemia control strategies in Nigeria, especially in the area of early detection of childhood anaemia.

Our findings confirm that the Haemoglobin Colour Scale showed good agreement with that obtained with the HemoCue assay. This is similar to what have been reported in other previous studies [7, 9]. The Haemoglobin Colour Scale has been found to be accurate in diagnosing severe and moderately severe anaemia in high-prevalence area in hands of community health workers in real-life field condition, after a brief training [20]. Furthermore, the sensitivity and specificity of the HCS in identifying anaemic children were impressive for a screening tool. Although efforts should be made to use WHO Hb values of 11g/dl as cutoff for anaemia rather than the 10g/dl classification according to the HCS instruction [13], interestingly, studies have shown that there is increase in the performance of the HCS as the haemoglobin level descends towards the range of severe anaemia cutoffs [11, 13, 15]. Since the specificity of the HCS at this low haemoglobin cutoff remains relatively high, coupled with the excessive morbidity and mortality risk associated with severe anaemia, the use of HCS can be recommended for the detection and treatment of severe childhood anaemia in primary care settings in resource poor countries that lack the sophisticated modern equipment for detecting anaemia.

The performance of clinical assessment of anaemia in children is less than optimal. This has been a common finding in many published studies [21–25] reporting clinical assessment of pallor. Despite this poor performance of clinical assessment of anaemia, it still remains the sole possible method of prompt evaluation for anaemia in primary healthcare facilities in developing countries. Our experience and that of Luby* et al.* [3] demonstrated that the act of clinical assessment of anaemia can be learned by parents/caregivers and other nonphysicians with minimal training. Their performance can be improved by providing varying colour charts that represent three broad categories: not anaemic, anaemic, and very anaemic. This will reduce the subjectivity associated with comparing parents' palms which oftentimes are anaemic themselves.

One limitation in this study was the small number of children that had severe (5) and moderate (29) anaemia compared to mild (80) anaemia [8], which limited the ability to test the accuracy of the detection of anaemia of HCS at different grades of anaemia using relativity observers curve. Furthermore, haemoglobin level of individuals varies over a very wide range which makes estimation of average, and thus application of the Bland-Altman analysis which is based on average or difference between a standard (HemoCue) and a reference value (HCS), not feasible. A future designed study that will ensure relatively equal number of children with different grades of anaemia will be required to determine the efficiency of HCS at varying levels of anaemia.

In conclusion, the HCS is reliable option for haemoglobin assay if access to HemoCue assay is limited. However, every case of childhood anaemia should be properly investigated doing a minimum of blood film examination, to detect the underlying cause(s) and treat, not just correct, the anaemia.

## Figures and Tables

**Table 1:** The performance of HCS in estimating haemoglobin using HemoCue assay as standard.

Variable	Performance of HCS	Performance of HCS	p-value
	Absolute figure (n)	Percentage (%)	
Anaemia detected (<11g/dl) (n=552)			
HemoCue	233	44.5	
HCS	262	50.0	0.25
Performance of HCS using HemoCue assay as Standard			
Total sample	524		
Number of anaemic children in the sample	233	44.5	
Sensitivity	233/262	89.1	
Specificity	261/290	90.2	
PPV	261/290	90.2	
NPV	261/290	90.2	
Performance of Clinical detection of anaemia using HemoCue as standard			
Total sample	524		
Number of anaemic children in the sample	233	44.4	
Sensitivity	168/233	72.1	0.03
Specificity	356/421	84.6	
Positive Predictive Value	168/233	72.1	
Negative Predictive Value	356/421	84.6	
			

**Table 2:** Proximity of the HCS to the HemoCue results obtained through cross-tabulation of both results.

	Proximity of Haemoglobin CS to HemoCue (g/dl)		
	± 1.0	1.1 – 2.0	>2.0
Haemoglobin Colour Scale (2.3%)	391 (74.7%)	121 (23%)	12

## Data Availability

The data used to support the findings of this study are included within the article.

## Abbreviations

HCS: Haemoglobin Colour Scale
LGA: Local government area
NPV: Negative predictive value
PPV: Positive predictive value
WHO: World Health Organization.

## Additional Points

In this study we observed that Haemoglobin Colour Scale (HCS) performance was close to that of HemoCue in detecting childhood anaemia, while clinical detection of childhood anaemia was suboptimal.
